# Problem-Based Versus Conventional Curricula: Influence on Knowledge and Attitudes of Medical Students Towards Health Research

**DOI:** 10.1371/journal.pone.0000632

**Published:** 2007-07-18

**Authors:** Hassan Khan, Ather M. Taqui, Muhammad Rizwanulhaq Khawaja, Zafar Fatmi

**Affiliations:** 1 Medical College, Aga Khan University, Karachi, Pakistan; 2 Department of Community Health Sciences, Aga Khan University, Karachi, Pakistan; London School of Hygiene and Tropical Medicine, Peru

## Abstract

**Background:**

Medical education curricula in developing countries should emphasize training in health research. This study compares the knowledge and attitudes towards health research between undergraduate medical students undertaking Problem Based Learning (PBL) versus conventional Lecture Based Learning (LBL).

**Methods:**

Two groups comprising 66 (LBL) and 84 (PBL) 4^th^ and 5^th^ year students from the medical college of Aga Khan University were administered a structured and validated questionnaire. Knowledge and attitudes of the two groups were recorded on a scale (graduated in percentages) and compared for statistical difference.

**Results:**

PBL students scored 54.0% while LBL students scored 55.5% on the knowledge scale [p-value; 0.63]. On the attitudes scale, PBL students scored 75.5% against a 66.7% score of LBL students [p-value; 0.021]. A higher proportion of PBL students (89%) had participated in research activities compared to LBL students (74%) and thus felt more confident in conducting research and writing a scientific paper.

**Conclusion:**

The PBL students showed slightly healthier attitudes towards health research compared to LBL students. Both groups demonstrated a similar level of knowledge about health research. The positive impact of the PBL curriculum on attitudes of medical students towards health research may help in improving research output from developing countries in future.

## Introduction

Health research training has been recognized as an important component of medical education. This is essential to help developing countries achieve self-reliance and implement evidence-based practice in healthcare [1]. Studies have shown that research experience during medical school is strongly associated with postgraduate research initiatives [2], [3] as well as future career achievements in academic medicine [4].

The development of research capacity is imperative at individual as well as institutional level to attain a sustainable improvement in health research [5]. To fill the void of physician-scientists in developing countries, initiatives are being taken to motivate medical students to undertake careers in research [6]. Various strategies are being employed for this purpose, e.g. mandatory and elective research assignments, student sections in indexed journals, organization of students' scientific conferences, molding of medical curriculum to integrate capacity building for research and holding of workshops on different aspects of conducting research [7]. A recent study conducted among Pakistani medical students reported that workshops about research skills could potentially improve the knowledge of medical students about health research [7]. The seven day workshop covered topics ranging from epidemiological study designs to manuscript writing. The same self-administered questionnaire as in the current study was distributed immediately before and after the workshop to assess its effectiveness in improving knowledge and attitudes towards health research.

The new trends in medical education focus on acquisition of skills, knowledge and attitudes rather than factual learning [8]. Problem analysis and decision making towards its solution are key skills in the practice of medicine and research [9]. The Problem Based Learning (PBL) approach is an educational strategy founded in the West, but has been increasingly popular in medical schools all over Asia [10]. The PBL curriculum aims to inculcate the above mentioned abilities in medical students and to promote self-directed lifelong learning. In the conventional lecture based curriculum (LBL), relatively little emphasis was placed on critical analysis, self-directed learning or problem-solving [9]. Instead, teaching was a teacher-directed process and the emphasis was on examination oriented learning of details. Therefore, in conventional curriculum students passively absorb information rather than actively acquire knowledge.

The demand to implement clinical and educational strategies based on evidence has increased in the past two decades [11]. This is especially true for the PBL curriculum because of its rapid adoption and popularity. However many studies indicate that its effectiveness is limited as an educational medium. Even though it may be slightly superior to the conventional curriculum in some aspects of medical education, it generally falls short of its claims [12]. With this background, this study was carried out to assess the influence of mode of curriculum (LBL vs. PBL) on the knowledge and attitudes of medical students towards health research.

## Methods

In Pakistan, medical college of the Aga Khan University is the first one to implement a PBL curriculum. First PBL class was admitted to the University in October 2002. Our objective was to compare the knowledge and attitudes of the senior medical students from PBL curriculum with their historical LBL cohort.

### Study Design & Sample:

Aga Khan University has recently shifted its curriculum from LBL to PBL. During June to July 2005, a cross-sectional knowledge and attitude survey was conducted among the last batches of 4^th^ and 5^th^ year medical students of Aga Khan University who were following LBL curriculum. The students were selected using convenience sampling. They were chosen according to the probability of proportionate sizes of the classes. Two years later, during March of 2007, the survey was repeated on the 4^th^ and 5^th^ year PBL medical students, with an identical tool and sampling method.

The sample size was calculated for two independent samples, assuming equal variances and difference of means to be 1.0. We required about 86 students from each group (LBL and PBL) at 95% confidence intervals and power of 80% after inflating the sample by 10% to account for non response.

### Outcome Variables, Questionnaire and Data Collection:

The information was collected on a pre-tested and structured questionnaire *(see Appendix S1 for questionnaire)* [13], adapted from the validated questionnaire designed by Vodopivec *et al* [14].

The questionnaire consisted of three parts namely, student's personal data, evaluation of student's knowledge and attitudes of science and scientific research. The demographic details of subjects included age, gender, and year of study and mode of learning at medical school. Mode of learning was classified into LBL and PBL system. Knowledge was assessed by ten multiple-choice questions. For each student, the percentage of correct answers was calculated as a representative of knowledge score. Six questions were asked to assess the attitudes of students towards health research and each answer was scored on a scale of 0.0 to 1.0. For each individual, score of individual questions was summed and then converted into percentage to represent the attitude score. The detailed questionnaire has been published in a previous study [13].

Questionnaires were handed over to subjects after obtaining their verbal informed consent. Participants were asked to return the completed questionnaire within two days. Those who failed to do so were not followed up and were taken as non-respondents. The final sample included 66 students from the LBL (77% response rate) and 84 students from the PBL (98% response) curriculum.

### Statistical Analysis:

The data was entered and analyzed in Statistical Package for Social Sciences 13.0 (SPSS, Inc., Chicago, IL, USA). Descriptive statistics were performed for mean scores and proportions. Student's *t*-test was used to compare the knowledge and attitude scores of LBL students against PBL student. The results were recorded as frequencies, means+standard deviations (SD) and p-values. For all purposes, a p-value of <0.05 was considered as the criteria of significance.

## Results

Mean age of students in both groups was 21.3±2.03 years. Of the 66 LBL students, 27.3% were females, while 36.9% of 84 PBL students were females. The mean age and gender distribution did not differ significantly amongst the two groups

The mean knowledge score of LBL students was 55.5% as compared to 54.0% score of PBL students [p-value; 0.63]. The mean attitudes score of the LBL students was 66.7% against a 75.5% score of PBL students [p-value; 0.021]. Table 1 shows the year-wise mean score of LBL and PBL groups.

**Table 1:** Pakistani medical students' knowledge and attitude towards research according to year and mode of learning at medical school

	Year	No.	Knowledge		Attitude	
			Mean±SD	p-value	Mean±SD	p-value
LBL	4^th^	32	58.4±17.3	0.630	60.7±27.2	0.021
	5^th^	34	52.6±12.9		66.2±18.6	
PBL	4^th^	43	52.3±20.1		77.5+14.6	
	5^th^	41	55.9±20.9		73.3+18.39	

LBL, Lecture-based learning; Problem-based learning; SD, Standard deviation

Table 2 demonstrates different responses for each attitude question amongst the two groups. PBL students were more likely to have participated in a research activity [p-value; 0.017] and felt more confident in conducting research and writing a scientific paper [p-value; 0.043].

**Table 2:** Comparison of responses to questions assessing attitude towards health research

Question	PBL	LBL	p-value
*Do you feel confident in interpreting and writing a research paper?*			
No	13 (15.5)	18 (27.3)	0.085
Yes, with assistance	57 (67.9)	43 (65.2)	
Yes, without assistance	14 (16.7)	5 (7.6)	
*Have you ever participated in a research project (apart from mandatory academic projects)?*			
Yes	75 (89.3)	48 (73.8)	0.017
No	9 (10.7)	17 (26.2)	
*Have you ever written a scientific paper?*			
Yes	47 (56)	30 (45.5)	0.25
No	37 (44)	36 (54.5)	
*Do you think undergraduate students should participate in research?*			
Yes	82 (97.6)	61 (92.4)	0.135
No	2 (2.4)	5 (7.6)	
*Do you think undergraduate students can plan and conduct a research project and write a scientific paper?*			
Yes	83 (98.8)	59 (90.8)	0.043
No	1 (1.2)	6 (9.2)	

## Discussion

The present study shows that the level of knowledge towards health research is similar among medical students studying in PBL and LBL curriculum. However, the attitudes towards health research were significantly better in the medical students studying in the PBL curriculum.

These results signify the positive impact of the PBL curriculum in improving the attitudes of medical students towards health research. However, the ability to improve the level of knowledge towards health research is similar for both educational curricula. A companion paper had showed that the knowledge and attitudes of the medical students towards health research were significantly better in the LBL group as compared to the PBL group [13]. This group of medical students was selected from the same university. However, the effect of ‘year of study’ was stated as a possible confounder, because the PBL and LBL groups were from different years of study. The current study reduces this selection bias by comparing PBL and LBL groups from the same years (year four and five). However, by comparing two groups of students in different points in time, other biases may be introduced. To the best of our knowledge, no other study has been conducted which compares the knowledge and attitudes towards health research among medical students in the two different curricula.

The medical students from both curricula are taught the theoretical essentials of research methodology, statistics and epidemiology during the first two years of their medical school. The way in which these topics are taught differs among the two curricula. The students from both curricula gain a substantial portion of their knowledge through lectures. However, the students in the PBL curriculum also have small group interactive discussions. Moreover, these regular discussions allow greater individual attention and participation and thus offer a unique opportunity to clarify concepts and discuss specific issues in details. These advantages were not available to the LBL group.

In the fourth year of medical school, students from both curricula conducted mandatory research in a dedicated two month period. During this rotation, students are involved in thinking of a research question, designing and implementing a protocol, collecting and analyzing data, writing a detailed report and giving presentations. Due to the concept of student-determined learning objectives in PBL curriculum, students have greater amount of free time on their hands. This might have allowed them to spend a greater amount of time in research activities as compared to LBL students. This difference might account for the difference in their attitudes towards health research. Several studies indicate that students in the PBL curriculum generally have healthier attitudes towards their curriculum, have more favorable moods and find learning environment more enjoyable than their LBL counterparts [12], [15]. These positive qualities may translate into better attitudes towards health research.

Both mandatory as well and extracurricular research during undergraduate years has a positive influence on the students' inclination towards research in later life [3]. The better attitudes of PBL students towards health research explain their greater involvement in research activities than LBL students. In this study, a significantly greater proportion of PBL students had participated in a research projects, other than the mandatory ones (see Table 2). This, in turn, has translated into a higher proportion of PBL students, being confident in conducting research and writing a scientific paper. The proportion of students having written a scientific paper was also higher among PBL group.

Studies have shown that students in the PBL curriculum use a wider range and number of resources for achieving learning goals and also feel more competent in information-seeking skills as compared to their LBL counterparts [15], [16]. These qualities are required in conducting research. However, these qualities should have a positive impact on both knowledge and attitudes towards health research. In our study, only the attitudes were significantly better in the PBL group. The similar level of knowledge can be explained by the fact that a large proportion of the theoretical essentials of research methodology and statistics were taught in a similar manner to both the groups in our study, and were assessed in the same way at the end of the second year.

Many studies which compare the ability of the two educational curricula to foster desirable qualities in medical students have been conducted. Examples of measured qualities include competence and understanding of basic and clinical sciences [11]. The present study has shown that the PBL curriculum is effective in improving the attitudes of medical students towards health research. At the same time, it maintains a high level of knowledge towards health research but does not improve it further when compared to the conventional LBL curriculum. These findings are encouraging because it means that the PBL curriculum can be used as a means to improve the current situation of health research in Pakistan and other developing countries. The current situation of health research in Pakistan is quite poor [17]. Improving attitudes of undergraduate medical students towards health research will nurture greater participation, development of a more robust research infrastructure and promotion of evidence-based medicine. Evidence-based medicine is crucial to improving health care in Pakistan. With rising health costs, local literature is important for facilitating evidence based and cost-effective decisions and thereby improving local clinical practice [18]. South Asia has a quarter of the world's population, a weak public sector health care, and an overwhelming disease burden, and thus the importance of health research here can not be stressed enough [5].

### Limitations

While interpreting the findings of this study, the following limitations should be kept under consideration. The two groups which were compared did not exist at the same point in time. The ideal setting for the study would have been two similar medical schools with groups of students enrolled in PBL and LBL simultaneously. This was not possible in our setting. The temporal difference between the two groups was two years during which the curriculum transition from LBL to PBL took place in the university. However, the two groups were still comparable because factors like teaching faculty at the university hospital and facilities available for health research remained fairly constant during the two years. If there had been changes in the above mentioned factors during the two years, it could have influenced the knowledge and attitude of the students. However, the only dramatic change which occurred in the two years was the change in curriculum; and that justifies the comparison of the two groups at different points in time.

The non-response rate in the LBL students was higher than the anticipated 10% which was used in sample size calculation. We believe that the primary reason for not responding was lack of time rather than the topic of the study. Due to the non-response rate of 23%, the actual sample collected was 10 less than the required sample size. This may have contributed to a selection bias.

This study was conducted at one private institution, which may be different other medical schools in Pakistan and elsewhere. This restricts the generalizability of the results.

In light of these limitations, the findings of the study must be interpreted in a prudent manner.

### Conclusion

In conclusion, medical students in the PBL and LBL curriculum have a similar level of knowledge towards health research. However, students in PBL curriculum have significantly healthier attitudes towards health research. This positive aspect of the PBL curriculum can be used to address the lack of quality health research in Pakistan and other developing countries. Further studies should be conducted to assess the long-term research activities of physicians who graduated through the PBL curriculum; to see if the higher levels of positive attitude towards health research actually translate into valuable health research output.

## Supporting Information

Appendix S1 Questionnaire
(0.03 MB DOC)

## References

[1] Scaria V (2004). Whisking Research into Medical Curriculum: The need to integrate research in undergraduate medical education to meet the future challenges.. Calicut Medical Journal 2004.

[2] Segal S, Lloyd T, Houts PS, Stillman PL, Jungas RL (1990). The association between students' research involvement in medical school and their postgraduate medical activities.. Acad Med.

[3] Reinders JJ, Kropmans TJ, Cohen-Schotanus J (2005). Extracurricular research experience of medical students and their scientific output after graduation.. Med Educ.

[4] Brancati FL, Mead LA, Levine DM, Martin D, Margolis S (1992). Early predictors of career achievement in academic medicine.. Jama.

[5] Sadana R, D'Souza C, Hyder AA, Chowdhury AM (2004). Importance of health research in South Asia.. Bmj.

[6] Bickel J, Morgan TE (1980). Research opportunities for medical students: an approach to the physician-investigator shortage.. J Med Educ.

[7] Khan H, Khawaja MR (2007). Impact of a workshop on the knowledge and attitudes of medical students regarding health research.. J Coll Physicians Surg Pak.

[8] (2005). Improving health by investing in medical education.. PLoS Med.

[9] Birgegard G, Lindquist U (1998). Change in student attitudes to medical school after the introduction of problem-based learning in spite of low ratings.. Med Educ.

[10] Khoo HE (2003). Implementation of problem-based learning in Asian medical schools and students' perceptions of their experience.. Med Educ.

[11] Sanson-Fisher RW, Lynagh MC (2005). Problem-based learning: a dissemination success story?. Med J Aust.

[12] Nandi PL, Chan JN, Chan CP, Chan P, Chan LP (2000). Undergraduate medical education: comparison of problem-based learning and conventional teaching.. Hong Kong Med J.

[13] Khan H, Khawaja MR, Waheed A, Rauf MA, Fatmi Z (2006). Knowledge and attitudes about health research amongst a group of Pakistani medical students.. BMC Med Educ.

[14] Vodopivec I, Vujaklija A, Hrabak M, Lukic IK, Marusic A (2002). Knowledge about and attitude towards science of first year medical students.. Croat Med J.

[15] Vernon DT, Blake RL (1993). Does problem-based learning work? A meta-analysis of evaluative research.. Acad Med.

[16] Coles CR (1985). Differences between conventional and problem-based curricula in their students' approaches to studying.. Med Educ.

[17] Rehan N (2003). Medical research in Pakistan.. J Coll Physicians Surg Pak.

[18] Aslam F, Qayyum MA, Mahmud H, Qasim R, Haque IU (2004). Attitudes and practices of postgraduate medical trainees towards research–a snapshot from Faisalabad.. J Pak Med Assoc.

