# Supplementary material for: Problem-Based Versus Conventional Curricula: Influence on Knowledge and Attitudes of Medical Students Towards Health Research
Source: PLoS One. 2007 Jul 18;2(7):e632. doi: 10.1371/journal.pone.0000632 (PMC1913552; doi:10.1371/journal.pone.0000632)
Supplement: Appendix S1 — Questionnaire (0.03 MB DOC) [file pone.0000632.s001.doc]

**Appendix S1**

**Knowledge and Attitudes about Health Research amongst Undergraduate Medical Students**

This questionnaire is designed to investigate some basic concepts of science and scientific research as well as attitudes of undergraduate students at The Aga Khan University. Please sign and fill in the following questionnaire if you wish to participate. A strict confidentiality will be maintained for all the information. We appreciate your cooperation.

Signatures----------------------

***Please circle only one response.***

**Age:** ____ Years

**Sex:** 1-Male 2-Female

**Year of study:** 1-First 2-Second 3-Third 4-Fourth 5-Fifth

**Mode of Curriculum**: 1) PBL 2) LBL

**Q1). How would you define the scientific hypothesis?**

A. a proposed idea or thought

B. an answer or solution to a question

C. an answer or solution to a question which has a capacity of verification or empirical demonstration

D. logical deduction of the premises that may or may not be verified empirically

**Q2). How would you define scientific theory?**

A) Speculation or assumption with no or insufficient evidence.

B) Scientific hypothesis that may be proven, but lacking evidence for verification.

C) Set of scientific knowledge on a given topic or area.

D) System of hypotheses logically connected to one another, with common background, some of which have been verified.

**Q3). How would you define the scientific truth?**

A. the truth that will be reached through scientific research

B. absolute truth

C. consensus of competent experts

D. fact that can be found in the textbooks

E. facts that your professors teach you

**Q4). The essential characteristic of science is:**

A. all scientific conclusions are temporary

B. scientific theory cannot merely explain natural phenomena, but must somehow also exert influence upon them

C. rather obvious scientific conclusion does not have to be testable

D. an experiment is not an objective model of the nature but serves as an introduction into real research of natural phenomena

E. some natural phenomena need not be measured but it suffices that a researcher notices them on time

**Q5). A scale from 1 to 5 (like grades on an examination) is called:**

A. ratio scale

B. nominal

C. ordinal

D. interval

E. it is not a scale

**Q6). Representativeness is a key characteristic of a:**

A. scientific paper

B. professional paper

C. scientific research

D. sample

E. population

**Q7). MEDLINE is:**

A. the first and best known “on-line” medical journal

B. international association of medical informaticians

C. printed form of the Excerpta Medica

D. abbreviation (acronym) that lists the parts of the research article

E. medical database

**Q8). In the previous year you have published a paper in the prestigious Journal of Immunology. Now you want to check the number of citations your paper has received. The best way to do it would be to search the:**

A. author index of the MEDLINE database

B. corporate index of the Science Citation Index database

C. author index of the Current Contents database

D. citation index of the Science Citation Index database

E. author index of the Science Citation Index database

**Q9).The part of a scientific paper is:**

A. author’s curriculum vitae

B. letter to the editor enclosed with the paper

C. description of the timeline

D. acknowledgment to persons who assisted you during the research

**Q10). All listed rules apply to the process of writing an**

**Introduction section of a scientific paper EXCEPT:**

A. clearly state why the research has been started

B. do not explain textbook facts

C. do not explain words from the title of the paper

D. make it longer rather than shorter

E. clearly define the question to which your research aims to provide an answer

**Q11). Do you feel confident in interpreting and writing a research paper? *(Please circle the appropriate number)***

A) No B) Yes, with assistance C) Yes, without assistance

**Q12).Have you ever participated in a research project (apart from mandatory academic projects)?**

A). Yes B). No

**Q13). Have you ever written a scientific paper?**

A). Yes B). No

**Q14). Do you think undergraduate students should participate in research? *(If No please do not proceed further)***

A). Yes B). No

**Q15). Do you think undergraduate students can plan and conduct a research project and write a scientific paper?**

A). Yes B). No

**Q16) If yes, encircle the appropriate**

A). only under supervision B) without supervision

**THE END**

**Initials of the Investigator____________________**
